# Effects of whole grain rye, with and without resistant starch type 2 supplementation, on glucose tolerance, gut hormones, inflammation and appetite regulation in an 11–14.5 hour perspective; a randomized controlled study in healthy subjects

**DOI:** 10.1186/s12937-017-0246-5

**Published:** 2017-04-21

**Authors:** Jonna C. Sandberg, Inger M. E. Björck, Anne C. Nilsson

**Affiliations:** Food for Health Science Centre, Lund University, SE-221 00 Lund, Sweden

**Keywords:** Rye, Glucose regulation, Dietary fiber, Whole grain, Dietary prevention, Gut hormones, Appetite regulation, Gut fermentation, Type 2 diabetes, Obesity

## Abstract

**Background:**

The prevalence of obesity is increasing worldwide and prevention is needed. Whole grain has shown potential to lower the risk of obesity, cardiovascular disease and type 2 diabetes. One possible mechanism behind the benefits of whole grain is the gut fermentation of dietary fiber (DF), e.g. non-starch polysaccharides and resistant starch (RS), in whole grain. The purpose of the study is to investigate the effect of whole grain rye-based products on glucose- and appetite regulation.

**Method:**

Twenty-one healthy subjects were provided four rye-based evening test meals in a crossover overnight study design. The test evening meals consisted of either whole grain rye flour bread (RFB) or a 1:1 ratio of whole grain rye flour and rye kernels bread (RFB/RKB), with or without added resistant starch (+RS). White wheat flour bread (WWB) was used as reference evening meal. Blood glucose, insulin, PYY, FFA, IL-6 as well as breath H_2_ and subjective rating of appetite were measured the following morning at fasting and repeatedly up to 3.5 h after a standardized breakfast consisting of WWB. *Ad libitum* energy intake was determined at lunch, 14.5 h after evening test and reference meals, respectively.

**Results:**

The evening meal with RFB/RKB + RS decreased postprandial glucose- and insulin responses (iAUC) (*P* < 0.05) and increased the gut hormone PYY in plasma the following morning 0–120 min after the standardized breakfast, compared to WWB (*P* = 0.01). Moreover, RFB increased subjective satiety and decreased desire to eat, and both RFB and RFB/RKB decreased feeling of hunger (AUC 0–210 min). All rye-based evening meals decreased or tended to decrease fasting FFA (*P* < 0.05, RFB/RKB: *P* = 0.057) and increased breath hydrogen concentration (0–120 min, *P* < 0.001). No effects were noted on energy intake at lunch or inflammatory marker IL-6 (0 + 180 min) after the rye-based evening meals, compared to WWB.

**Conclusion:**

Whole grain rye bread has the potential to improve cardiometabolic variables in an 11–14.5 h perspective in healthy humans. The combination RFB/RKB + RS positively affected biomarkers of glucose- and appetite regulation in a semi-acute perspective. Meanwhile, RFB and RFB/RKB improved subjective appetite ratings. The effects probably emanate from gut fermentation events.

**Trial registration:**

The study was registered at: ClinicalTrials.gov, register number NCT02347293 (www.clinicaltrials.gov/ct2/show/NCT02347293). Registered 15 January 2015.

## Background

The worldwide increase in prevalence of obesity-related metabolic diseases such as the metabolic syndrome, cardiovascular disease and type 2 diabetes is predicted to continue [1]. These diseases are lifestyle-related and therefore to a large extent preventable. In this respect, increased intake of whole grain (WG) has shown health benefits. Thus epidemiological data show that WG intake is associated with reduced risk of type 2 diabetes [2], cardiovascular disease [3], and also a decreased risk of obesity and weight gain [4]. At least parts of the beneficial effects of WG are suggested to emanate from mechanisms originating from gut fermentation of indigestible carbohydrates, i.e. dietary fiber (DF) components such as non-starch polysaccharides (NSP) and resistant starch (RS). Previously it was shown that intake of whole kernel barley or rye products improved markers of glucose regulation [5–9], and increased gut hormones involved in appetite- and metabolic regulation (GLP-1 and/or PYY) [5–7], 11–14 h after ingestion in healthy subjects. The effects were suggested to originate from gut fermentation of the DF components.

WG flour based products are usually rich in NSP, whereas the RS level is lower in comparison to WG kernel based products, making the total amounts of DF inferior in flour based products. It has previously been suggested that products made from WG flour with a low content of RS did not beneficially affect metabolic variables as oppose to WG kernel based products rich in RS. Consequently, opposite to what was observed after kernel based barley products, it was shown that barley flour porridge did not result in beneficial effects on glucose regulation in a 10.5–12.5 h perspective [10]. The lower concentrations of RS in WG flour may be compensated for e.g. by adding ungelatinized RS (RS2) from high-amylose maize (HAMRS2). RS2 is a fermentable source of DF previously shown to lower acute insulin response (0–420 min after intake) in healthy humans when added to a standardized breakfast and lunch [11]. In addition, adding RS2 to a barley DF supplemented white wheat bread (WWB) improved glucose tolerance at breakfast, 10.5–12.5 h after intake, compared to WWB without supplementation [7]. However, to the best of our knowledge, there is no information regarding the effect of supplementation of RS2 to WG rye products on metabolic parameters in healthy subjects within a short time frame. Rye is a rich source of indigestible carbohydrates mainly in the form of arabinoxylans, fructans, cellulose, β-glucan and lignin [12] but depending on the botanical integrity, i.e. proportion of kernels to flour, the RS content may vary.

The aim of this study was to investigate the potential impact of WG rye products (bread), varying in botanical structure of rye and RS content, on cardiometabolic risk markers and appetite regulation, in a time perspective of 11–14.5 h after intake. For this purpose, four WG rye breads with different flour to kernel ratio and supplementation of commercial RS2 were provided to healthy subjects as evening meals. A white wheat bread was included as a reference evening meal. The rye-based test products and the reference product were consumed in the evening, in a randomized crossover study design, and test markers in blood were determined the next morning prior and following a standardized breakfast to measure glucose regulation (glucose and insulin), gut hormones (peptide YY (PYY)), blood lipids (free fatty acids (FFA)) and inflammatory tonus (interleukin 6 (IL-6)). Additionally, breath hydrogen (H_2_) excretion was measured as a marker of gut fermentation, and subjective appetite ratings (hunger, satiety, and desire to eat) were registered. Finally, *ad libitum* energy intake was registered at a lunch provided 3.5 h after the standardized breakfast.

## Materials and methods

### Ethical statement

The study was registered at ClinicalTrials.gov (NCT02347293). Approval of the study was given by the Regional Ethical Review Board in Lund, Sweden (Reference 2013/241).

### Subjects

Twenty-four healthy volunteers, 11 men and 13 women were enrolled in the study. Three of the subjects, 1 man and 2 women, did not complete all five visits. The twenty-one volunteers that completed the study and were included in the statistical evaluation were aged 25.3 ± 3.9 years (mean ± standard deviation (SD)), with normal body mass indices (BMI) (mean ± SD = 22.7 ± 2.3 kg/m^2^). The inclusion criteria were age between 20 – 35 years, BMI between 19 and 25 kg/m^2^, non-smoker and no known metabolic disorders, food allergies or consumption of any specific diet (e.g. vegetarian or vegan diet). Recruitments of test subjects were ongoing between January 21 to March 3, 2015, and the experimental work was finished by May 22, 2015. Prior inclusion, each subject was given a full written as well as an oral explanation of the purpose and procedure of the study, and written informed consent was obtained from each subject. All test subjects were informed of the possibility of withdrawing from the study at any time they desired.

### Test products and reference product

The study included four different rye-based test products, presented as cereal dry matter (DM): 100% whole grain (WG) rye flour based bread (**RFB**)**,** 50% WG rye flour and 50% rye kernel based bread (**RFB/RKB**), 75% WG rye flour based bread with 25% added high-amylose maize flour (Hi-Maize® 260, including 60% RS2) (**RFB + RS**), and 43% WG rye flour and 43% rye kernel based bread with 14% added Hi-Maize® 260 (**RFB/RKB + RS**). A 100% DM white wheat flour based bread (**WWB**) was included as reference product. The proportions of ingredients in the rye-based test products and reference product WWB are displayed in Table 1. The size of the test portions, to be consumed in the evening, was based on 50 g available starch. Both the rye flour and rye kernels (separate commercial blends) were generously provided by Lantmännen Cerealia (Järna, Sweden), Hi-Maize® 260 (60% RS2 and 40% digestible starch) was kindly provided by Kåkå (Lomma, Sweden) and commercial white wheat flour was obtained from Kungsören AB (Järna, Sweden).

**Table 1:** The proportions of cereal ingredients in the rye-based test- and WWB reference evening meals^1^

	Test products				
	RFB	RFB/RKB	RFB + RS	RFB/RKB + RS	WWB
	*% dry matter*				
Whole grain rye flour	100	50	75	43	**-**
Rye kernels	**-**	50	**-**	43	**-**
HAMRS2	**-**	**-**	25	14	**-**
White wheat flour	**-**	**-**	**-**	**-**	100

^1^ Data presented as % dry matter. *RFB* rye flour based bread, *RFB/RKB* rye flour and kernel based bread, *RS* resistant starch, *RFB + RS* RFB with added RS, *RFB/RKB + RS* RFB/RKB with added RS, *WWB* white wheat flour based reference bread, *HAMRS2* high-amylose maize resistant starch type 2

#### RFB

600 g whole grain rye flour, 6 g yeast (Jästbolaget AB, Sollentuna, Sweden) and 6 g NaCl were mixed for 1 min in a food processor (Electrolux Assistent AKM3110W) and then 400 g lukewarm water was added and an additional 4 min of mixing was done. The dough was proofed at room temperature in a bread pan for 30 min. Baking was performed in a house-hold oven at 200 °C with steam the first 15 min and then the bread was covered with aluminum foil the last 45 min (total baking time 60 min).

#### RFB/RKB

300 g rye kernels were boiled in 250 g salted water (6 g NaCl) for 30 min and then set to cool in room temperature for 30 min. All water was absorbed into the kernels when cooked. 300 g whole grain rye flour and 6 g yeast (Jästbolaget AB, Sollentuna, Sweden) were added to the kernels and were mixed for 4 min in a food processor (Electrolux Assistent AKM3110W). The dough was proofed at room temperature in a bread pan for 30 min. Baking was performed in a house-hold oven at 200 °C with steam the first 15 min and then the bread was covered with aluminum foil the last 45 min (total baking time 60 min).

#### RFB + RS

The bread was baked according to the same procedure as RFB, except that 200 g Hi-Maize was mixed together with the other dry ingredients (600 g whole grain rye flour, 6 g yeast and 6 g NaCl).

#### RFB/RKB + RS

The bread was baked according to the same procedure as RFB/RKB, except that 100 g Hi-Maize was mixed together with the 300 g cooled rye kernels along with the other dry ingredients (300 g whole grain rye flour and 6 g yeast).

After baking, the bread was cooled for 3 h, then placed in a plastic bag overnight. In the morning the bread was sliced into portions, wrapped in aluminum foil, put into plastic bags and stored in a freezer (−20 °C). Portion sizes are presented in Table 2.

**Table 2:** Portion size and carbohydrate composition of the test- and reference products respectively^1^

Product	Portion size	Starch			NSP		Total DF	DF components	
		Total	Available	RS	Insoluble	Soluble		Fructan	Arabinoxylan
*% dry matter*									
RFB		59.3	56.0	3.4	13.9	5.1	22.4	3.3	7.3
RFB/RKB		58.2	51.2	6.9	14.6	4.4	25.9	3.9	7.0
RFB + RS		67.4	54.2	13.2	15.9	3.5	32.6	2.5	5.0
RFB/RKB + RS	-	71.5	58.9	12.7	14.3	4.1	31.1	3.2	6.3
WWB	-	80.2	78.5	1.7	3.4	0.9	6.0	0.5	0.5
*g/portion*									
RFB	187.2	53.0	50.0	3.0	11.0	4.1	18.1	3.0	6.5
RFB/RKB	184.0	56.8	50.0	6.8	12.5	3.7	23.0	3.8	6.8
RFB + RS	168.4	62.1	50.0	12.1	11.9	2.9	26.9	2.3	4.6
RFB/RKB + RS	157.9	60.8	50.0	10.8	9.9	3.1	23.8	2.7	5.3
WWB	122.0	51.0	50.0	1.1	2.0	0.5	3.6	0.3	0.3

^1^ Data are presented as means. Available starch is calculated as difference between total starch [14] and RS [15]. Values of total starch are based on means of 2 replicates, RS means of 6 replicates and DF are based on means of 2 replicates. Insoluble and soluble DF were determined gravimetrically according to Asp et al. (1983) [16]. The fructan content was analyzed in duplicates using the enzymatic/spectrophotometric AOAC method 999.03 [17]. Analysis of arabinoxylans was performed in duplicates using the Uppsala method [18] and was corrected for the amount of arabinogalactan present by assuming 0.69 ratio between arabinose and galactose [19]. Total DF include RS, and insoluble and soluble NSP. *RFB* rye flour based bread, *RFB/RKB* rye flour and kernel based bread, *RS* resistant starch, *RFB + RS* RFB with added RS, *RFB/RKB + RS* RFB/RK with added RS, *DF* dietary fiber, *NSP* non-starch polysaccharides, *WWB* white wheat flour based reference bread

#### WWB

The WWB was baked in a home baking machine (Tefal home bread model nr. 573102; Menu choice, program 2 [white bread, 1000 g, quick (time2:32)]) according to a standardized procedure. The bread ingredients consisted of 540 g white wheat flour, 360 g water, 4.8 g dry yeast and 4.8 g NaCl. After cooling, the crust was removed and the bread was sliced and portions were wrapped in aluminum foil, put into plastic bags and stored in a freezer (−20 °C). Portion sizes are presented in Table 2. WWB was used both as reference evening meal and included in the standardized breakfast, see below.

The test subjects stored the bread products in their freezer during the study period. At the day of consumption, they were instructed to take a portion from the freezer in the morning and thaw it at ambient temperature, still wrapped in aluminum foil and in the plastic bag. Optional amounts of water, coffee or tea (without milk and/or sugar) were allowed to be consumed with the test- and reference products.

### Standardized breakfast

The standardized breakfast consisted of 122.0 g WWB, corresponding to 50 g available starch, and 200 ml of water. The breakfast bread was baked according the same standardized procedure as WWB, reference product.

### *Ad libitum* lunch

To evaluate *ad libitum* energy intake, the test subjects were instructed to serve themselves with lunch consisted of fried Swedish hash, i.e. diced potato, meat and onion (Felix Krögarpytt Klassisk), from an oven sheet. The lunch was generously provided by Orkla Foods Sverige AB (Malmö, Sweden). According to previous analysis by Johansson et al. (2013) the nutritional content is 234 kcal/100 g of Swedish hash, whereof the proportion of macronutrients (% DM) is 38.5% carbohydrates, 13.9% protein and 27.5% fat [6]. 100 ml of water was provided just before the lunch was served.

### Study design and procedure

The study included five bread products: four test products (RFB, RFB/RKB, RFB + RS and RFB/RKB + RS) and one reference product (WWB) which were consumed as evening meals (09.00 pm) in a randomized cross-over study design, with approximately 1 week in between each test product. After this meal, the test subjects were fasting until the standardized breakfast was served the following morning at the research unit Food for Health Science Centre, Lund University. Number of participants (*n* = 21, subjects including in the statistical evaluation) starting with respective product: RFB (*n* = 4), RFB/RKB (*n* = 5), RFB + RS (*n* = 3), RFB/RKB + RS (*n* = 5) and WWB (*n* = 4).

During the day prior to each experimental day, the subjects were encouraged to standardize their meal pattern and to maintain their regular eating habits with the exception to avoid food rich in DF. They were also instructed to avoid alcohol and excessive physical exercise during the day prior to the experimental day. No antibiotics or probiotics were allowed within 2 weeks before or during the study period. To facilitate the standardization of meal patterns prior to each experimental day during the study period, the test subjects were requested to provide a meal record from the day before each experimental day. The test subjects were in addition given a list of foods rich in DF to simplify the instructions regarding avoidance of DF the day before the experimental day. At the experimental days, the subjects arrived to the research unit at 0730 am. Fasting capillary blood samples were collected, and appetite and breath H_2_ excretion were registered before the breakfast. A standardized breakfast was served at 0800 am and finished within 15 min. At time point 210 min after commencement of the breakfast, *ad libitum* lunch was served. The test subjects served themselves food from an oven sheet with a surplus of food and were instructed to eat until they felt pleasantly full and to obtain the same degree of induced satiation (i.e. the process that results in termination of eating [13]) at every experimental day. To attain this, they were informed that they were allowed to have a second serving and also to leave food on their plate if necessary. The amount (weight) of lunch consumed was registered. The test subjects were told to sustain a low physical activity during the experimental day (4.5 h).

### Chemical analyses of evening meals and standardized breakfast

The test products and breakfast were analyzed with respect to total starch [14], RS [15], NSP (soluble and insoluble) [16], fructans [17] and arabinoxylans [18]. Information regarding the characterization of carbohydrates in the test and reference meals is provided in Table 2. Before analysis of total starch and NSP, the bread products were air dried and milled (IKA A11 basic mixer model A11 B, Germany). RS in all test products were analyzed on products as eaten. The available starch content was calculated by subtracting RS from total starch. The enzymatic/spectrophotometric AOAC method 999.03 was performed to analyze the fructo-oligosaccharides and inulin (in the following referred to as fructans) content, in duplicate, in the test products using the enzyme assay kit K-FRUC (Megazyme, Bray, Ireland). To avoid possible interference of raffinose-series oligosaccharides the analysis included treatment with α-galactosidase [17]. Arabinoxylans were determined by analyzing the monomeric composition in the isolated dietary fiber residue from the test products using the Uppsala method [18]. The amount of arabinoxylan content was calculated from the xylose, galactose and arabinose, and corrected for the amount of arabinogalactan present by assuming ratio 0.69 between arabinose and galactose [19].

### Physiological test parameters

Fasting and postprandial measurements were performed at all five visits. Finger-prick capillary blood samplings were applied for determination of blood glucose (HemoCue®B-glucose, HemoCue AB, Ängelholm, Sweden). Additional capillary blood samples were collected to determine serum (s-) insulin, s-FFA, s-IL-6 and plasma (p-) PYY. Breath hydrogen was measured as an indicator of gut fermentation activity, using an EC 60 gastrolyzer (Bedfont EC60 Gastrolyzer, Rochester, England). Pre- and post-breakfast subjective appetite sensations (satiety, hunger and desire to eat) were obtained using a 100 mm Visual Analogue Scale (VAS). Breath H_2_ and VAS ratings for subjective appetite (hunger, satiety and desire to eat) were obtained at fasting (time 0 min) and at 15, 30, 45, 60, 90, 120, 180 and 210 min after commencing the breakfast. Blood glucose was determined on similar times points, excluding 210 min, and s-insulin was collected at all time points except 15 and 210 min. Plasma PYY was determined at time 0 min and at 120 min after breakfast. Serum IL-6 was obtained at time 0 min and at 180 min. Serum FFA was determined at time 0 min.

*Serum insulin* was determined with a solid phase two-site enzyme immunoassay kit (Insulin ELISA 10-1113-01, Mercodia AB, Uppsala, Sweden), *s-FFA* concentrations with an enzymatic colorimetric method using a 96 well microplate (NEFA C, ACS-ACOD method, WAKO Chemicals GMbH, Germany). The quantitative determination of *s-IL-6* was performed with an enzyme immunoassay (Human IL-6 HS600B, R&D Systems, Abingdon, UK) and the concentrations of *PYY,* both PYY (3–36) and PYY (1–36), in plasma were determined with a competitive enzyme immunoassay (Human PYY EIA YKO8O, Yanaihara Institute Inc. Shizuoka, Japan).

### Calculations and statistical methods

Data are expressed as means ± SEM. Statistical evaluations involving glucose and insulin concentrations are based on incremental changes from fasting concentrations. The absolute values are used in the statistical calculations of the rest of the test variables. The incremental area- and area under the curve (iAUC and AUC, respectively) were calculated for each subject and evening meal, using the trapezoid model. iAUC or AUC were used in the statistical evaluation of results regarding blood glucose, insulin and parameters for appetite sensations. Incremental peak (iPeak) concentrations were calculated for glucose and insulin as individual maximum postprandial increase from the baseline. GraphPad Prism (version 6, GraphPad Software, San Diego, CA, USA) was used for graph plotting and calculation of AUC.

Investigations with respect to significant differences in test variables depending on the different evening meals were assessed with ANOVA (general linear model) followed by Dunnett’s multiple comparison procedure, using results after the reference product WWB as a control. The data was analyzed in MINITAB Statistical Software (release 17; Minitab, Minitab Inc, State College, PA, USA). The significance level was set at *P*-values < 0.05. In the cases of unevenly distributed residuals (tested with Anderson-Darling and considered unevenly distributed when *P* < 0.05), a Box Cox transformation was performed on the data prior to the ANOVA analysis. Differences between the products at different time points during the experimental day were evaluated using a mixed model (PROC MIXED in SAS release 9.4; SAS Institute Inc, Cary, NC, USA) with repeated measures and an autoregressive covariance structure. Randomization of the consumption order of test products was performed using a random number function in Microsoft Excel 2013 (Washington, USA). If a result of a test variable from a test subject was missing for the reference product, the test subject was excluded from the statistical calculations of that specific test variable. If a result of a test variable from a test subject was missing for one of the test products, the test subject’s results for the remaining products will be included in the statistical calculations of that specific test variable. For breath H_2_, where the variation in the concentration changed scarcely over time, a weighted mean was produced by calculating a mean for equal time intervals (one mean per hour) over the test period, and then a mean for the different hours was calculated and used in the statistical analysis.

### Power calculation

Primary outcome measure was change in blood glucose incremental area under the curve (iAUC) 0–120 min after the standardized breakfast. Number of participants required for the study was determined in MINITAB, using previous results from a study including single evening test meals consisting of rye kernels and the effects the following morning on glucose incremental area under the curve 0–120 min (iAUC) [5]. Assuming a 44 mmol · min/L (25%) difference between consumption of WWB compared to rye-based test products the previous evening, and a SD of 60 mmol · min/L, with α = 0.05 and 1 − β = 0.8, a minimum number of 17 participants were required (two-tailed test). However, due to lack of information from previous similar study designs including different rye flour based products and including drop-out margin, we recruited 24 test subjects. Three of the subjects dropped out of the study, which resulted in 21 subjects remaining in the statistical evaluation.

## Results

### Glucose and insulin

**Fig. 1:**
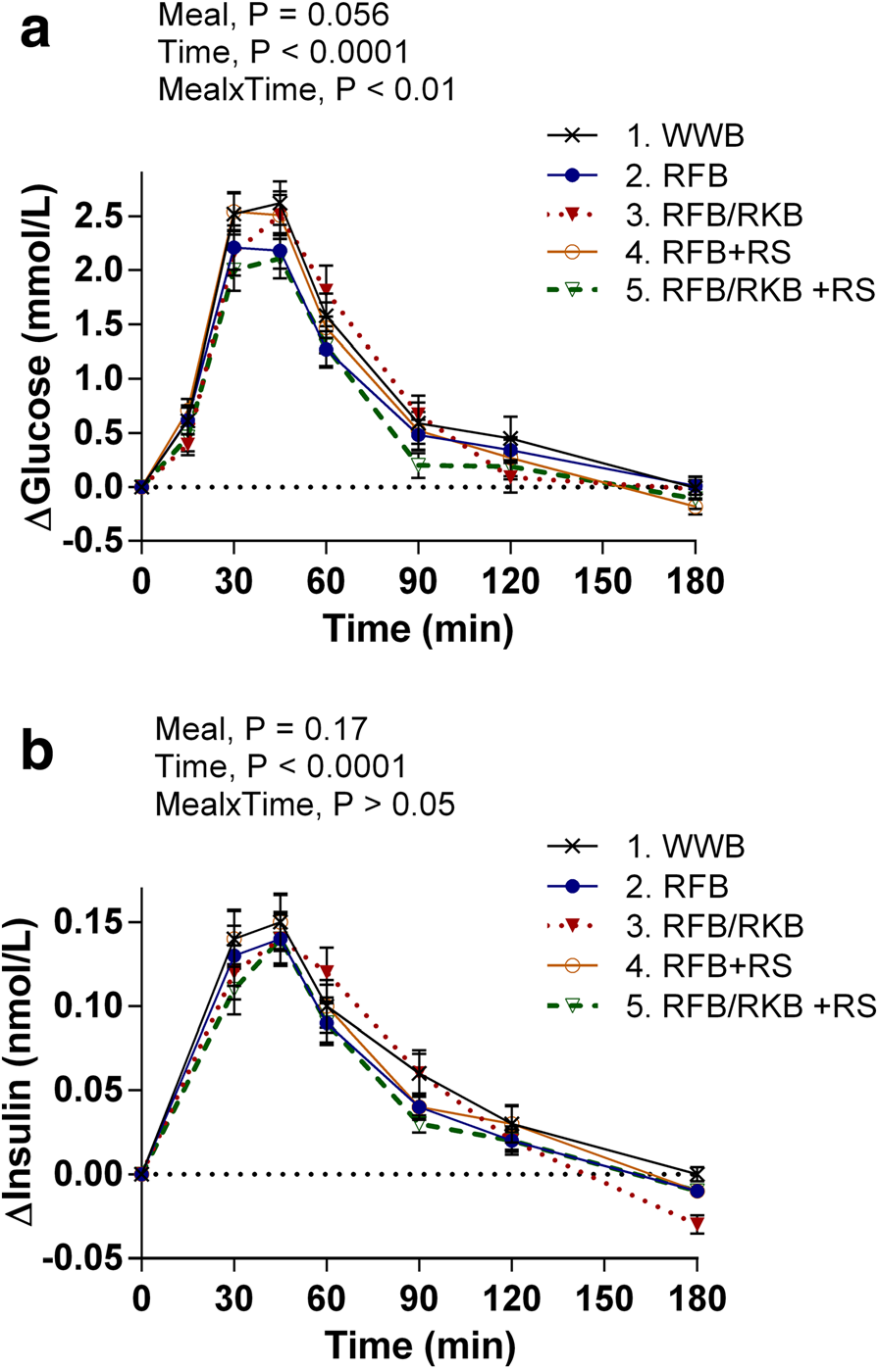
Incremental blood glucose- and serum insulin concentrations after a standardized breakfast following the evening meals Changes (Δ) in mean incremental concentrations of blood glucose (**a**) and serum insulin (**b**) from fasting concentrations in the morning after a standardized breakfast, 11–14 h following the rye-based test- or WWB reference evening meals. Values are means ± SEM. Repeated measures; mixed model in SAS. RFB, rye flour bread; RFB/RKB, 50/50 rye flour and rye kernel bread; + RS, added resistant starch; WWB, white wheat bread

Compared to the WWB reference evening meal, the evening meal consisting of RFB/RKB + RS decreased (iAUC 0–120 min) responses for glucose (−27%) and insulin (−21%) in the morning following the standardized breakfast, see Table 3 and Fig. 1. A strong tendency to decrease glucose iPeak concentrations was observed after the standardized breakfast following intake of RFB/RKB + RS evening meal (−17%, *P* = 0.057), compared to WWB, see Table 3 and Fig. 1. The evening meal with RFB, in comparison to the WWB evening meal, resulted in a non-significant trend towards a decrease (−19%, *P* = 0.100) in postprandial glucose responses (iAUC 0–120 min) to the standardized breakfast. There were no significant differences in fasting values after test products compared to reference product for neither glucose nor insulin concentrations.

**Table 3:** B-glucose, s-insulin, s-FFA, p-PYY and s-IL-6 concentrations 11–14 h following test- and reference evening meals^1^

	WWB	RFB		RFB/RKB		RFB + RS		RFB/RKB + RS	
			%Δ^**2**^		%Δ^**2**^		%Δ^**2**^		%Δ^**2**^
Glucose, Fasting^3^ *(mmol/l)*	5.4 ± 0.1	5.3 ± 0.1	−1	5.4 ± 0.1	0	5.4 ± 0.1	1	5.3 ± 0.1	−2
Glucose, iAUC 0–120 min^3^ *(mmol · min/l)*	153.0 ± 13.2	123.6 ± 10.8	−19	140.2 ± 13.8	−8	138.8 ± 15.0	−9	112.1 ± 10.6	−27*
Glucose, iPeak ^3^ *(mmol/l)*	2.9 ± 0.2	2.6 ± 0.2	−11	2.8 ± 0.2	−3	2.8 ± 0.2	−2	2.4 ± 0.2	−17†
Insulin, Fasting^3^ *(nmol/l)*	0.034 ± 0.002	0.038 ± 0.003	13	0.037 ± 0.003	10	0.036 ± 0.003	6	0.033 ± 0.002	−3
Insulin, iAUC 0–120 min^3^ *(nmol · min/l)*	9.88 ± 1.21	8.50 ± 0.91	−14	9.52 ± 1.13	−4	9.40 ± 1.00	−5	7.86 ± 0.78	−21*
Insulin, iPeak^3^ *(nmol · min/l)*	0.16 ± 0.02	0.16 ± 0.02	0	0.16 ± 0.02	0	0.17 ± 0.02	1	0.15 ± 0.02	−8
FFA, Fasting *(mmol/l)*	0.65 ± 0.04^3^	0.54 ± 0.04^4^	−17*	0.54 ± 0.04^4^	−17†	0.54 ± 0.04^3^	−17*	0.54 ± 0.03^3^	−17*
PYY, Fasting^4^ *(ng/ml)*	0.50 ± 0.09	0.54 ± 0.10	7	0.53 ± 0.09	6	0.53 ± 0.10	6	0.59 ± 0.09	17*
PYY, Mean 0 + 120 min^4^ *(ng/ml)*	0.50 ± 0.09	0.53 ± 0.10	5	0.52 ± 0.09	5	0.53 ± 0.09	6	0.58 ± 0.09	15**
IL-6, Fasting *(pg/ml)*	1.08 ± 0.21^3^	0.81 ± 0.06^3^	−25	0.93 ± 0.12^3^	−15	1.00 ± 0.12^4^	−7	0.82 ± 0.09^3^	−25
IL-6, Mean 0 + 180 *(pg/ml)*	1.33 ± 0.22^4^	1.04 ± 0.15^5^	−22	1.02 ± 0.14^4^	−23	1.20 ± 0.16^5^	−9	1.02 ± 0.12^4^	−23

^1^ Data are presented as means ± SEM. *Different from WWB *P* < 0.05; **P = 0.01, †*P* = 0.057 (ANOVA followed by Dunnett’s test). ^2^ The percentage change is calculated as the difference from the WWB. ^3^ *n* = 21, ^4^ *n* = 20, ^5^ *n* = 19 healthy subjects. *RFB* rye flour bread, *RFB/RKB* 50/50 rye flour and rye kernel bread, *+ RS* added resistant starch, *iAUC* incremental area under curve, *iPeak* incremental peak

### s-FFA

The fasting concentrations of FFA are presented in Table 3 and were significantly decreased after test evening meals with RFB, RFB + RS and RFB/RKB + RS, compared to WWB (−17% for all, *P* < 0.05). Additionally, a tendency towards decreased FFA concentrations was shown after RFB/RKB (−17%, *P* = 0.057).

### s-IL-6

No differences were observed in the concentrations of IL-6 depending on the evening meal, see Table 3.

### p-PYY

**Fig. 2:**
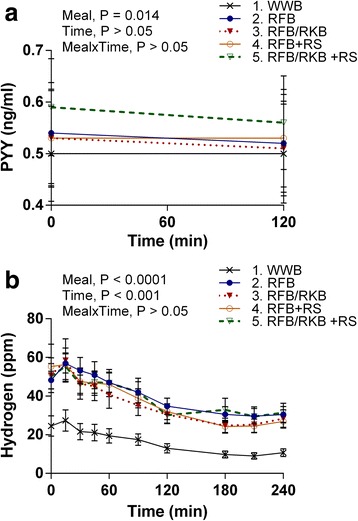
Concentrations of plasma PYY and breath hydrogen after a standardized breakfast following the evening meals Mean concentrations of plasma PYY (**a**) and breath hydrogen excretion (**b**) in the morning after a standardized breakfast, 11–13 h respectively 11–14.5 h following the rye-based test- or WWB reference evening meals. Values are means ± SEM. Repeated measures; mixed model in SAS. RFB, rye flour bread; RFB/RKB, 50/50 rye flour and rye kernel bread; + RS, added resistant starch; WWB, white wheat bread

P-PYY response at the standardized breakfast is presented in Table 3 and Fig. 2. In comparison with the reference WWB, the evening meal comprising RFB/RKB + RS resulted in an increased p-PYY concentration at fasting (+17%, *P* < 0.05) as well as an increased postprandial mean concentration including time points 0 and 120 min (+15%, *P* = 0.01).

### Breath H_2_

The response in breath H_2_ is presented in Table 4 and Fig. 2. In comparison to WWB, all rye-based evening meals resulted in increased breath H_2_ levels at fasting, that remained increased after the standardized breakfast (*P* < 0.001).

**Table 4:** Breath hydrogen excretion, subjective appetite ratings and voluntary energy intake (lunch), following the evening meals^1^

	WWB	RFB		RFB/RKB		RFB + RS		RFB/RKB + RS	
			%Δ^**2**^		%Δ^**2**^		%Δ^**2**^		%Δ^**2**^
Hydrogen, Fasting^3^ *(ppm)*	24.5 ± 5.3	48.3 ± 4.5	97***	50.8 ± 9.0	107***	55.3 ± 11.5	126***	50.2 ± 6.6	105***
Hydrogen, Mean 0–210 min *(ppm)*	16.2 ± 2.9^3^	40.4 ± 4.0^3^	150***	35.9 ± 5.5^3^	122***	37.6 ± 4.3^3^	133***	34.8 ± 4.5^4^	115***
Satiety, Fasting^3^ *(mm)*	29.2 ± 4.5	35.0 ± 5.3	20	37.9 ± 5.9	30	29.6 ± 4.3	2	33.3 ± 6.1	14
Satiety, AUC 0-210 min *(mm · min)*	9590 ± 930^5^	11640 ± 950^5^	21**	11390 ± 1110^6^	19	10070 ± 930^5^	5	10400 ± 910^6^	8
Hunger, Fasting^3^ *(mm)*	61.2 ± 4.4	52.5 ± 6.4	−14	51.7 ± 6.0	−15	58.6 ± 5.7	−4	58.3 ± 5.7	−5
Hunger, AUC 0–210 min *(mm · min)*	10400 ± 950^5^	8800 ± 880^7^	−15*	8680 ± 1070^4^	−17*	10090 ± 910^5^	−3	9340 ± 840^6^	−10
Desire to eat, Fasting^3^ *(mm)*	67.1 ± 5.0	62.9 ± 5.8	−6	58.7 ± 5.7	−13	68.3 ± 5.5	2	63.4 ± 6.7	−6
Desire to eat, AUC 0–210 min *(mm · min)*	11360 ± 1020^5^	9070 ± 1030^5^	−20**	9750 ± 1170^4^	−14	11110 ± 1020^5^	−2	11450 ± 1090^6^	1
Energy intake, Lunch^3^ *(kcal)*	689 ± 87	657 ± 74	−5	644 ± 73	−7	649 ± 61	−6	667 ± 70	−3

^1^ Data are presented as means ± SEM. *Different from WWB *P* < 0.05; **P < 0.01, ***P < 0.001 (ANOVA followed by Dunnett’s test). ^2^ The percentage change is calculated as the difference from the WWB. ^3^ *n* = 21, ^4^ *n* = 18, ^5^ *n* = 20, ^6^ *n* = 17, ^7^ *n* = 19 healthy subjects. *RFB* rye flour bread, *RFB/RKB* 50/50 rye flour and rye kernel bread, *+ RS* added resistant starch, *AUC* area under curve

### Subjective appetite ratings and energy intake

**Fig. 3:**
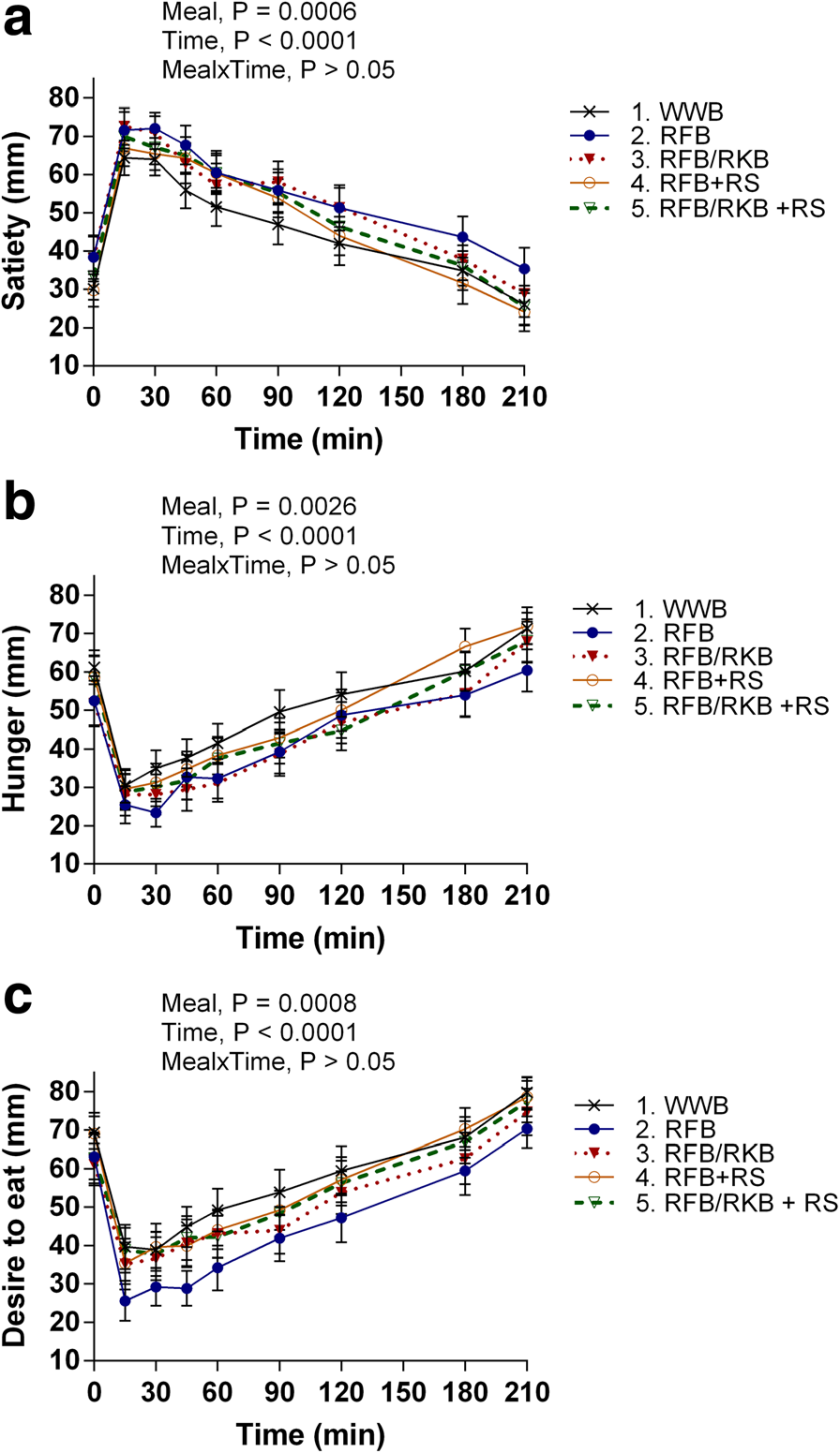
Subjective appetite ratings after a standardized breakfast following rye-based test- or WWB reference evening meal Data is presented as mean ± SEM of subjective appetite ratings (VAS) of satiety (**a**), hunger (**b**) and desire to eat (**c**) during the following 3.5 h after standardized breakfast in the morning, 11**–**14.5 h after intake of a rye-based evening meal or a WWB reference evening meal. Repeated measures; mixed model in SAS. RFB, rye flour bread; RFB/RKB, 50/50 rye flour and rye kernel bread; + RS, added resistant starch; WWB, white wheat bread

The data for subjective appetite ratings are shown in Table 4 and Fig. 3. The RFB evening meal significantly increased the feeling of satiety (21%, *P* < 0.01) and decreased feelings of hunger (−15%, *P* < 0.05) and desire to eat (−20%, *P* < 0.01) during the experimental day, 0–210 min after the standardized breakfast (AUC), compared to WWB. In addition, the evening meal RFB/RKB decreased the hunger sensation during the experimental day (AUC 0–210 min, −17%, *P* < 0.05). No significant differences were seen in the energy intake at lunch depending on the evening meal.

## Discussion

It has previously been shown that certain kernel based cereal products, e.g. barley kernel products, and more recently also rye kernel products, beneficially affected cardiometabolic test variables 11–16 h [6] or 10.5–13.5 h [5] after intake, in comparison to a flour based product (WWB). The effects were proposed to emanate from gut fermentation of the mixture of DF in the intact kernels, i.e. NSP and RS. In the present study, it was hypothesized that similar metabolic benefits could be achieved also with bread containing rye flour or rye flour mixed with rye kernels by simulating the DF composition and amount in the intact kernels, through supplementation with commercial RS (HAMRS2) to compensate for the loss of RS (RS1) in the milling process. Previous inclusion of HAMRS2 in the diet was shown to beneficially impact insulin demand up to 7 h after ingestion in healthy humans and to reduce energy intake during 24 h, compared to a low-RS diet [11]. RS has also been shown to stimulate secretion of GLP-1 and PYY in rats fed a RS diet for 10 days [20].

The hypothesis was confirmed, with respect to the RFB/RKB product with and without RS supplementation. Consequently, postprandial glucose regulation was improved and plasma PYY levels increased in a time perspective of 11–13 h after intake of the RFB/RKB + RS, compared to WWB. No such effects were seen after the RFB/RKB without RS supplementation. The results are thus in support of the importance of RS components, and/or the specific mixture of NSP and RS in kernels on metabolic regulation. However, independently of the RS content, all rye-based test products resulted in a decrease in fasting FFA concentrations the following morning compared to the WWB evening meal, with potential benefits on insulin sensitivity. An improved glucose regulation and decreased concentrations of FFA were recently observed in the morning when rye kernel based bread was included as an evening meal [5]. Increased levels of FFA have been observed in type 2 diabetes patients compared to those with normal glucose tolerance [21] and were associated with impaired insulin signaling [22]. Hence, the present results indicate that rye kernels have beneficial semi-acute effects on glucose regulation and insulin sensitivity.

Previously, intake of rye kernels were shown to increase plasma levels of SCFA 10.5 h after intake [5], and as judged from the increased levels of breath H_2_ following the rye-based evening meals, the metabolic benefits seen in the present work are most likely emanating from gut fermentation. Substrate-induced increase in SCFA concentrations in the gut may potentially have beneficial implications on appetite- and metabolic regulation since SCFA has been suggested to stimulate the L-cells to secrete GLP-1 and PYY [23], and in addition increase the uptake of circulating FFA [24]. In the present study, plasma levels of SCFA were not determined. However, RFB/RKB + RS significantly increased the concentrations of plasma PYY 11 and 13 h after intake. In fact, the other rye-based products also tended to increase the PYY concentrations in plasma. It has previously been shown that intravenous infusion with PYY in lean or obese subjects decreased the food intake with 31% respectively 30%, at an *ad libitum* buffet served after 2 h, compared to infusion with saline [25], indicating an anti-obesogenic potential of PYY. A rye kernel based evening meal has previously shown to increase both PYY and GLP-1 the following morning [5]. In this context, although GLP-1 concentrations were not measured in this study due to small quantities of plasma via capillary sampling, it might be anticipated that an increase in PYY would indicate a potential increase also in GLP-1 since PYY is co-released together with GLP-1 after a meal [26]. These gut hormones have been shown to have anti-diabetic properties [27] and could partially explain the improved glucose regulation after intake of the rye-based test products.

Despite the significantly increased PYY levels in the present study, the effects on appetite variables and energy intake at lunch did not reach statistical significance after intake of RFB/RKB + RS. Instead, the non-supplement products, i.e. 100% flour based rye bread (RFB) and the RFB/RKB bread, which induced less increased PYY levels compared to RFB/RKB + RS, showed the most pronounced beneficial effects on subjective appetite ratings. In accordance, it has been reported that PYY levels do not always correlate with subjective appetite [28]. However, this does not preclude the potential role of PYY in the appetite regulation but that the mechanisms remain to be elucidated.

Although no significant effects on energy intake at lunch depending on the previous evening meals could be detected in this study, there was a non-significant decrease in energy intake after RFB/RKB evening meal (−7%) in comparison to the WWB. Consequently, this is in accordance with results after boiled rye kernels in a previous study with similar study design [29]. A decrease in energy intake (−12%) in this time perspective has previously been shown also after intake of barley kernel based products [6], indicating potential anti-obesogenic properties of certain fermentable DF. The energy content at the standardized breakfast in the current study, 273 kcal, can be considered to be low (kcal content obtained from [30]), and it can be speculated that, at least for some of the test subjects, the low energy content at breakfast could have interfered with the possibility to differentiate voluntary energy chosen at the subsequent lunch. The previous over-night studies ([6, 29]), including barley or rye as an evening meal, showed effects on voluntary energy intake at lunch after a more realistic and high caloric breakfast composed of either an *ad libitum* breakfast [6] or a breakfast with e.g. butter, cheese, jam (and a pastry) [29]. In this context it is worth to consider that these breakfasts probably closer resemble the test subjects’ normal diets compared to the breakfast included in the current study (WWB and water).

It has previously been shown that an evening meal including WWB supplemented with a mix of RS (similar to the RS product used in the present study) and NSP (from barley) in proportions similar to the composition and content of indigestible carbohydrates in intrinsic barley kernels, significantly lowered the glucose response the following morning. Further it was observed that supplementation of a WWB with RS or DF separately did not improve glucose tolerance the next morning, compared to un-supplemented WWB [7, 9]. This indicates that the combination of RS and NSP is necessary to mediate benefits on glucose tolerance in a time perspective of approximately 10–14 h, such as from the evening meal to a subsequent standardized breakfast. As expected, based on the chemical analysis it was confirmed that RFB and RFB/RKB contained less RS compared to the RS supplemented products. In the present study the RS supplementation of the RFB/RKB product seems to be important with respect to effects on glucose regulation and release of PYY. However, supplement with RS in the form of HAMRS2 did not improve other test variables included in the study. Probably both the total amounts- and composition of DF components are important. The rye portion in the RFB + RS was replaced with 25% HAMRS2 flour (DM), and in the RFB/RKB + RS the replacement with HAMRS2 flour was 14%. The HAMRS2 flour product included, in addition to RS (60%), a considerable proportion of available starch (40%). Thus, the lack of benefits with the HAMRS2 supplement to RFB, i.e. RFB + RS, can possibly be due to an important loss of rye DF due to dilution of the rye dry matter when replacing rye flour with HAMRS2. A loss of DF was confirmed in the chemical analysis and showed that both RFB + RS and RFB/RKB + RS contained lower concentrations of soluble DF, and decreased levels of fructans and arabinoxylans, compared to the breads without RS supplementation. Thus, since the only known difference between the ingredients in rye-based products is the addition of RS, it can be suggested that the comparatively high concentrations of soluble fiber in the un-supplemented RFB and RFB/RKB could have contributed to the beneficial effects observed on appetite ratings after these products. This is to the best of our knowledge the first study that observes significant difference in appetite ratings 11–14.5 h after intake of WG rye flour product. These effects have previously only been observed in acute studies when serving WG rye flour bread as breakfast [31, 32].

A limitation in the study was the small volumes of blood samples obtained, due to capillary blood sampling instead of venous blood sampling. Consequently, it was not possible to investigate additional parameters of inflammatory variables and gut hormones. Another potential study limitation was the relative low number of subjects and the low energy intake at breakfast when investigating subjective appetite sensations during the experimental day and voluntary food intake at lunch. Nevertheless, the appetite ratings and energy registration from the present study indicate that rye-based products, in particular the combination of WG rye flour and rye kernels (RFB/RKB), have the potential to lower energy intake at the subsequent day, but further studies are necessary to confirm this.

## Conclusion

The present study showed that addition of RS to a rye bread based on 50% intact kernels and 50% WG flour (DM) beneficially impact metabolic parameters in an 11–14 h perspective, as demonstrated by improved postprandial glucose regulation, decreased concentration of FFA at fasting and increased levels of PYY. In addition, it was shown that WG rye flour bread contributed to increased sensation of satiety and that WG rye flour bread solely or combined with rye kernels lowered perceived hunger sensations, 11–14.5 h after intake. The findings suggest that WG rye has anti-diabetic and anti-obesogenic potential. More studies are needed to further evaluate the metabolic effects of inclusion of commercially available RS fractions in different food matrices.

## Abbreviations

AUC: Area under curve
b-: Blood-
BMI: Body mass index
DF: Dietary fiber
DM: Dry matter
f-: Fasting-
FFA: Free fatty acids
GLP-1: Glucagon-like peptide-1
H_2_: Breath hydrogen
HAMRS2: High-amylose maize resistant starch type 2
iAUC: Incremental area under curve
IL-6: Interleukin-6
iPeak: Incremental peak
NSP: Non-starch polysaccharides
PYY: Peptide YY
RFB: Rye flour bread
RKB: Rye kernel bread
RS: Resistant starch
RS2: Resistant starch type 2
SCFA: Short chain fatty acids
SD: Standard deviation
SEM: Standard error mean
VAS: Visual analogue scale
WG: Whole grain
WWB: White wheat bread
